# Cancer-Associated Thrombosis: Beyond Clinical Practice Guidelines—A Multidisciplinary (SEMI–SEOM–SETH) Expert Consensus

**DOI:** 10.1055/s-0038-1675577

**Published:** 2018-11-05

**Authors:** Vanessa Pachón, Javier Trujillo-Santos, Pere Domènech, Enrique Gallardo, Carmen Font, José Ramón González-Porras, Pedro Pérez-Segura, Ana Maestre, José Mateo, Andrés Muñoz, María Luisa Peris, Ramón Lecumberri

**Affiliations:** 1 Department of Oncology, Hospital Universitario Ramón y Cajal, Madrid, Spain; 2 Department of Internal Medicine, Hospital Universitario Santa Lucía, Cartagena, Spain; 3 Thrombosis and Haemostasis Unit, Hospital Universitario Bellvitge, L'Hospitalet de Llobregat, Catalonia, Spain; 4 Department of Oncology, Parc Taulí Hospital Universitari, Institut d'Investigació i Innovació Parc Taulí I3PT, Universitat Autònoma de Barcelona, Sabadell, Spain; 5 Department of Medical Oncology, Hospital Clinic, Barcelona, Spain; 6 Hematology Service, Hospital Universitario de Salamanca-IBSAL, Salamanca, Spain; 7 Department of Oncology, Hospital Clínico San Carlos, Madrid, Spain; 8 Department of Internal Medicine, Hospital del Vinalopó, Elche, Spain; 9 Hematology Service, Hospital Santa Creu i Sant Pau, Barcelona, Spain; 10 Department of Oncology, Hospital Universitario Gregorio Marañón, Madrid, Spain; 11 Department of Internal Medicine, Hospital Provincial de Castellón, Castellón, Spain; 12 Hematology Service, Clínica Universidad de Navarra, IDISNA, CIBER-CV, Pamplona, Spain

**Keywords:** cancer, venous thrombosis, pulmonary embolism, prophylaxis, treatment

## Abstract

Despite the growing interest and improved knowledge about venous thromboembolism in cancer patients in the last years, there are still many unsolved issues. Due to the limitations of the available literature, evidence-based clinical practice guidelines are not able to give solid recommendations for challenging scenarios often present in the setting of cancer-associated thrombosis (CAT). A multidisciplinary expert panel from three scientific societies—Spanish Society of Internal Medicine (SEMI), Spanish Society of Medical Oncology (SEOM), and Spanish Society Thrombosis and Haemostasis (SETH)—agreed on 12 controversial questions regarding prevention and management of CAT, which were thoroughly reviewed to provide further guidance. The suggestions presented herein may facilitate clinical decisions in specific complex circumstances, until these can be made leaning on reliable scientific evidence.

## Introduction

Cancer patients have a high risk of venous thromboembolism (VTE), sometimes being the first manifestation of a so-far hidden malignancy. Mechanisms leading to thrombus formation in cancer patients are incompletely understood, although the risk may be partly influenced by the antineoplastic therapy itself. The management of cancer-associated thrombosis (CAT) frequently represents a challenge for clinicians due to very poor or lack of evidence regarding common daily practice scenarios that have also been poorly addressed by most currently available evidence-based guidelines. 1 2 3 4 5

This work arises from a joint initiative of a multidisciplinary panel of experts under the auspice of the Spanish Society of Internal Medicine (SEMI), Spanish Society of Medical Oncology (SEOM), and Spanish Society of Thrombosis and Haemostasis (SETH), who leaned on literature to reach consensus on controversial issues aimed to guide clinicians to manage complex, albeit not uncommon, situations related to CAT, until further evidence supporting or discouraging the proposed recommendations becomes available. Our aim is not to produce another evidence-based guideline on the field but to provide useful advice for scenarios that clinicians involved in CAT have to face without the support of unequivocal strong evidence-based recommendations. Due to the specific wording of the questions, most of them have not been previously approached elsewhere.

## Methods

In the first meeting, the whole panel agreed on 12 specific controversial questions that were to be addressed. The topics were identified by a recent Delphi study and completed by own experience. 6 The questions were distributed among four teams of three experts, including one member of each society (i.e., three questions per team). For every topic, the available literature (from previous guidelines to small studies) was reviewed and an initial consensus was reached inside each working team leading to a proposal of suggestions for the assigned questions. In the final meeting, the whole panel discussed all the proposals until agreement was reached. An executive summary is presented in Table 1 , also including the most relevant literature for each topic.

**Table 1:** Summary of recommendations

Question	Background	Suggestions
**Prophylaxis**		
1. In ambulatory cancer patients, should thrombotic risk be evaluated using a risk score to decide on the use of antithrombotic prophylaxis?	• The accuracy of Khorana, Vienna-CATS, PROTECHT, or CONKO scores is limited. 10 11 12 • COMPASS, ONKOTEV, and ONCOTHROMB require validation. 13 14 16 • Some tumor-specific scores have been recently developed showing promising results. 17 18	• Thrombotic risk should be evaluated, but not only by using the Khorana's risk score. • Attention has to be paid to new scores with improved predictive ability, although validation is required.
2. In cancer patients who are hospitalized for an acute medical illness, when is pharmacological antithrombotic prophylaxis contraindicated *?*	• Hospitalized cancer patients have a high risk of VTE and, whenever possible, preventive measures have to be implemented. 1 2 3 4 5 • However, studies that weigh the risk–benefit balance in this specific population are lacking. • Therefore, safety must be specially considered in the clinical decision-making process.	• Absolute contraindications of pharmacological prophylaxis: - Recent bleeding in CNS; active major bleeding; platelet count <20 × 10 ^9^ /L. • Relative contraindications: - Relevant chronic bleeding (duration >48 h); initial period of postneurosurgery; spinal or intracranial lesions; platelet count 20–50 × 10 ^9^ /L; drug-related platelet dysfunction or uremia; underlying coagulopathy. • Wait 12 h after last prophylactic-dose LMWH administration for lumbar puncture or spinal anesthesia. • In case of contraindication, apply physical antithrombotic measures. • Thromboprophylaxis is not required in cancer patients hospitalized exclusively to receive oncologic treatment (except in case of immobilization).
**Initial treatment**		
3. Must LMWH dose be modified in cancer patients with acute VTE treated with antiangiogenic drugs?	• Most clinical trials assessing antiangiogenic drugs excluded anticoagulated patients. • Indirect information can be obtained from some bevacizumab studies. 25 26 27 28 29 30 31 32 33	• In general, the LMWH dose should not be modified in patients developing a VTE event while on antiangiogenic treatment. • Special caution is required in case of CNS involvement. • Resumption of the antiangiogenic therapy (if indicated) should be delayed at least 2 wk after starting LMWH.
4. In patients with CAT requiring surgery or invasive procedure, when should the placement of an inferior vena cava filter be considered?	• Results from studies on the use of IVCF in cancer patients with VTE are controversial. 34 35 36 37 38 39 40 • Consensus exists on its use in patients with PE or DVT when ACG is contraindicated, especially in the first weeks after VTE. 41 42 43 44 • Concern about early and delayed adverse effects associated with IVCF is increasingly growing. 41 44 45 46 47	• Use of IVCF is suggested in cancer patients with acute lower limb proximal DVT/PE, who require a procedure that contraindicates ACG, particularly in the first 2–4 wk after the thrombotic episode. • After the first 2–4 wk, use IVCF only if proximal DVT persists. • While IVCF remains inserted, if possible, administer LMWH at least at prophylactic doses. • Remove IVCF and restart full ACG as soon as the cause that led to placement is resolved.
**Long-term treatment**		
5. In patients with CAT that require extended anticoagulant therapy beyond 6 mo, what is the optimal dose if LMWH is maintained?	• Guidelines recommend maintaining ACG beyond 6 mo in cases of active cancer and/or ongoing chemotherapy, although optimal drugs and doses are not specified. 44 48 • Evidence on LMWH doses to use beyond 6 mo is scarce. • An observational 49 and two single-arm prospective studies, DALTECAN 50 and TICAT (451), provide useful information. • DOACs may be an alternative for selected patients with low risk of gastrointestinal bleeding and drug interactions. 54 56	• Decide dose according to characteristics of patient, of the disease and its treatment, and of the VTE event: - Patient ▪ Full doses: obesity; thrombophilia; immobilization; venous insufficiency; varicose veins and low bleeding risk. ▪ Intermediate/prophylactic doses: renal failure or thrombocytopenia, or/and high bleeding risk. - Tumor ▪ Full doses: metastatic disease or locoregional disease with vessel compression; tumors with high thrombotic risk. ▪ Intermediate/prophylactic doses: tumors with lower thrombotic risk. - VTE event ▪ Full doses: life-threatening symptomatic PE, recurrent VTE, postthrombotic syndrome. ▪ Intermediate/prophylactic doses: incidental PE; isolated LL DVT; CVC-associated thrombosis; recurrent SVT. ▪ Dose increased by 25%: VTE recurrence in spite of appropriate LMWH.
6. Should anticoagulant treatment be prolonged beyond 3–6 mo in cancer patients with CVC-DVT, when the central venous line is maintained? Is LMWH prophylaxis indicated in patients with previous CVC-DVT if a new CVC has to be inserted?	• Current recommendations are not uniform across different guidelines and not supported by evidence of sufficient quality. 43 44 57 58 59 • If the CVC is maintained beyond the first 3–6 mo of anticoagulation after a CVC-DVT, the scenario could be considered as a VTE secondary to a persistent risk factor.	• After 3–6 mo, if bleeding risk is not high, prolong ACG using intermediate or prophylactic doses of LMWH until CVC removal. • If a new CVC is inserted in a patient with previous history of CVC-DVT, use prophylaxis with LMWH for 30 d. Consider longer periods depending on bleeding risk and patient's preferences.
**Treatment of VTE in complex scenarios**		
7. How should CAT be treated in cases of primary or secondary central nervous system involvement?	• Anticoagulation is effective, and usually well tolerated, in patients with gliomas 62 or cerebral metastases. 63 Some data favor treatment modifications under certain circumstances. • Brainstem hemorrhages are particularly serious.	• In general, use LMWH according to standard guidelines. • The following exceptions are made: - In secondary CNS involvement due to melanoma or kidney cancer, if VTE is not severe, reduce LMWH dose by 25–50%. - In patients with brainstem glioma, initially reduce LMWH dose by 25–50%, until local control of the disease is achieved.
8. Should incidental splanchnic venous thrombosis be treated?	• In a prospective registry of splanchnic VT, not limited to cancer patients, recurrences were more frequent in male patients with incidental thrombosis and shorter time on ACG. 66 67 • While on anticoagulant treatment, in patients with incidentally diagnosed splanchnic VT, the rate of major bleeding did not exceed that of recurrent thrombosis, although specific results in cancer patients are unknown. • Individualization is suggested according to chronic/nonchronic nature of thrombus. 42 68	• Start ACG treatment unless there is a formal contraindication. • Individualize in case of: - Data suggestive of chronic thrombosis. - Isolated thrombosis of intrahepatic portal segmental branch. • ACG should be maintained for at least 3 mo.
9. In cancer patients with acute VTE, what platelet count threshold would imply modifications in the LMWH dose? Can platelet transfusions avoid LMWH dose reductions?	• Full-dose ACG is accepted with platelet counts >50 × 10 ^9^ /L. Controversy arises with lower values. • There is reticence about recommendation of transfusing platelets to reach >50 × 10 ^9^ /L to maintain therapeutic LMWH doses [ISTH and Canadian Consensus 41 69 74 ]: it is complex and is associated with risk of adverse effects. 70 71 • Others support dynamic strategies of ACG dose reduction, 73 or platelet transfusion with lower thresholds (<20 × 10 ^9^ /L). 71	• With counts ≥50 × 10 ^9^ /L, maintain full doses of LMWH. • With counts between 20 and 50 × 10 ^9^ /L, reduce LMWH 50%. • With counts ≤20 × 10 ^9^ /L - If >30 d since VTE diagnosis, withhold ACG. - If <30 d since VTE diagnosis, transfuse platelets to maintain counts >20 × 10 ^9^ /L, and use intermediate LMWH doses. • Consider IVCF in the acute phase of VTE (especially when thrombocytopenia is thought to last >5 d) in: - Patients with counts ≤20 × 10 ^9^ /L. - Patients with counts 20–50 × 10 ^9^ /L and low cardiopulmonary reserve.
10. In patients with acute CAT requiring anticoagulant treatment and who were under antiplatelet therapy, when should the latter be maintained?	• ACG with VKA prevents coronary disease progression, and ischemic stroke in AF patients. 76 • The combined use of antiplatelet drugs with ACG treatment does not always improve ischemic events prevention and increases hemorrhagic risk. 77 78 • The use of VKA together with two antiplatelet drugs involves a high hemorrhagic risk, but may be justified for short periods in situations of high thrombotic risk. 79 • In patients with thrombosis associated with a MPN, the benefit of combined aspirin plus ACG is probably overweighed by the increased risk of bleeding. 81 82 83	• In cancer patients on ACG treatment for VTE, maintain antiplatelet drugs only in exceptional situations of markedly elevated risk of coronary events. • Maintenance of antiplatelet treatment in patients who are going to start anticoagulant therapy for CAT is justified in case of recent (<1 y) ACS event or placement of a coronary stent. • In patients with acute CAT carrying stents in other vascular beds, maintenance of antiplatelet treatment while on anticoagulant therapy should be decided in a case-by-case basis.
**Laboratory**		
11. In cancer patients treated with LMWH, when should anti-factor Xa activity be monitored?	• In studies with LMWH for the treatment of CAT, no relevant accumulation over time was observed. 55 84 85 • However, there are situations where pharmacokinetics of LMWH may be affected. 87 • Thrombocytopenia does not alter LMWH pharmacokinetics. • An association between anti-Xa activity and either clinical efficacy or hemorrhagic risk has not been demonstrated. 89	• In patients with CAT, routine monitoring of anti-Xa activity is not required. • In cases of creatinine clearance <30 mL/min, extreme body weight, or pregnancy, LMWH dose adjustment according to peak anti-Xa activity is suggested. • Monitoring anti-Xa in patients with thrombocytopenia or with other hemorrhagic risk factors is not suggested. The LMWH dose should not rely on this variable. • Monitoring anti-Xa activity is not suggested for prophylactic doses of LMWH.
12. Should thrombophilia study be performed in patients with CAT?	• Thrombophilic abnormalities have little influence on clinical decisions on ACG for VTE. 43 91 92 • In the cancer setting, initial ACG treatment for CAT is similar in patients with thrombophilia, and ACG duration is mainly influenced by cancer status. 42 43 44 91	• In patients with CAT, do not routinely investigate the presence of thrombophilia.

Abbreviations: ACG, anticoagulation/anticoagulant; ACS, acute coronary syndrome; AF, atrial fibrillation; anti-Xa, anti-factor Xa; CNS, central nervous system; CAT, cancer-associated thrombosis; CVC, central venous catheter; CVC-DVT, deep venous thrombosis associated with central venous catheter; DVT, deep venous thrombosis; ISTH, International Society on Thrombosis and Haemostasis; IVCF, inferior vena cava filter; LL, lower limbs; LMWH, low-molecular-weight heparin(s); MPN, myeloproliferative neoplasm; PE, pulmonary embolism; SEMI, Spanish Society of Internal Medicine; SEOM, Spanish Society of Medical Oncology; SETH, Spanish Society of Thrombosis and Haemostasis; SVT, superficial vein thrombosis; VKA, vitamin K antagonists; VTE, venous thromboembolic event/venous thromboembolism.

Notes: Twelve experts from the SETH, SEOM, and SEMI formed four teams of three, which included one member of each society. Each team elaborated initial consensus statements on three different questions. After a subsequent discussion with the participation of the whole panel of experts, a final consensus was reached for each one of the 12 proposed questions, all of which are controversial because of the scarce solid literature available about them.

## Results

### Question 1: In Ambulatory Cancer Patients, Should the Thrombotic Risk Be Evaluated Using a Risk Score to Decide on the Use of Antithrombotic Prophylaxis?

#### Background

To date, the only validated prediction model of CAT is the Khorana risk score. 7 However, in recent years several studies in different types of cancer suggest that this should not be the only tool to select candidate patients for antithrombotic prophylaxis in the outpatient setting 8 9 10 11 :

- A recent assessment questions the usefulness of the Khorana score, Vienna-CATS prediction model, PROTECHT score, or CONKO score for this aim. 12
- Two recent models, COMPASS 13 and ONKOTEV, 14 which rely on clinical parameters only, seem to overcome the predictive ability of the Khorana score. At 6 months, the area under the curve (AUC) of receiving operating characteristics of the COMPASS risk assessment model was 0.85. The ONKO-TEV score showed an AUC at 3 months of 71.9 versus 57.9% with the Khorana score. However, validation is still required.
- The addition of some biomarkers such as D-dimer or genomic risk profiles may help improve the usefulness of VTE risk scores. 15 16
- A different strategy, based on tumor-specific assessment models, has been developed. The Throly score, developed for patients with lymphomas, showed high negative predictive value (NPV; 97%), although the positive predictive value (PPV) was 15%. Another new risk model for prediction of VTE in gynecological cancer patients has also shown promising results. 17 18

Currently, routine thromboprophylaxis in ambulatory cancer patients is not recommended. Better tools to stratify VTE risk are needed to favor a primary prevention strategy in ambulatory cancer patients.

#### Suggestions

- Assessment of thrombotic risk in cancer patients on ambulatory treatment is suggested, with the purpose of identifying those who would, theoretically, benefit more from antithrombotic prophylaxis.
- Although Khorana's risk score is the only validated prediction model, it should not be the only tool used to select the patients who will receive ambulatory antithrombotic prophylaxis. Bleeding risk factors also have to be considered.
- New predictive models including biomarkers such as D-dimer or genomic risk profile, or tumor-specific scores, may help improve risk stratification.

### Question 2: In Cancer Patients Who Are Hospitalized for an Acute Medical Illness, When Is Pharmacological Antithrombotic Prophylaxis Contraindicated?

#### Background

Current guidelines agree that cancer patients who are hospitalized for any complication related to their clinical condition are at very high VTE risk, recommending pharmacologic prophylaxis with low-molecular-weight heparin (LMWH), unless contraindicated. 1 2 3 4 5 However, studies that specifically address the risk–benefit of thromboprophylaxis in cancer inpatients are lacking. In fact, recommendations are based on the results of trials whose cohorts consisted of heterogeneous groups of medical patients, among which cancer patients were underrepresented (5–15%). 19 20 21 22 23 A recent meta-analysis restricted to the cancer subgroup of the aforementioned studies did not confirm a positive effect of thromboprophylaxis. 24

On the other hand, cancer patients also exhibit an increased bleeding tendency and are considered as a high-risk population for hemorrhages. Therefore, a careful benefit–risk balance for each individual patient is advisable. Although validated tools to assess bleeding risk in cancer patients are lacking, several circumstances imply a contraindication for pharmacologic thromboprophylaxis.

#### Suggestions

- Settings where primary thromboprophylaxis with LMWH is contraindicated for cancer inpatients:
  - Absolute contraindications:
    ▪ Recent bleeding in the central nervous system.
    ▪ Active major bleeding.
▪ Thrombocytopenia <20 × 10 ^9^ /L.
  - Relative contraindications:
    ▪ Clinically relevant chronic bleeding, lasting for more than 48 hours.
    ▪ Initial period of postneurosurgery (48–72 hours).
    ▪ High bleeding risk–associated spinal or intracranial lesions (e.g., melanoma or kidney metastases).
    ▪ High risk of falls.
▪ Thrombocytopenia 20 × 10 ^9^ to 50 × 10 ^9^ /L.
    ▪ Severe platelet dysfunction.
    ▪ Underlying coagulopathy.
    ▪ Lumbar puncture or spinal anesthesia (procedures should be delayed 12 hours after last prophylactic LMWH dose).
- When pharmacological thromboprophylaxis is contraindicated, alternative use of mechanical measures is suggested.
- Thromboprophylaxis with LMWH is not necessary in patients admitted to hospital for scheduled oncological treatment who are not immobilized.

### Question 3: Must the LMWH Dose Be Modified in Cancer Patients with Acute VTE Receiving Antiangiogenic Drugs?

#### Background

Patients on anticoagulant therapy were explicitly excluded from most clinical studies with antiangiogenic drugs. 25 26 Furthermore, in those studies that allowed participation of anticoagulated patients, vitamin K antagonists (VKA) instead of LMWH (drug of choice for CAT treatment) were mostly used. Certain evidence arises from several clinical trials evaluating bevacizumab:

- Observational prospective or phase IV studies.

The BEAT 27 and the BRITE 28 studies provided comparative analyses on the incidence of severe bleeding (SB [grades 3–5]) between anticoagulated and non-anticoagulated bevacizumab-treated patients. Both studies showed higher SB rates among anticoagulated patients (4.3 vs. 2.4% in BEAT and 6.0 vs. 2.2% in BRITE, respectively). In contrast, in the SAIL study the SB rate in patients under anticoagulant therapy was null, compared with 4% in the overall cohort. 29

- Clinical trials allowing anticoagulant therapy.

In the pivotal trial, the proportion of patients who maintained the study treatment after suffering a VTE event and starting anticoagulant therapy was 6.5% in the bevacizumab arm and 3.4% in the placebo arm. 26 SB episodes were experienced by 3.8 and 6.7% of those patients, respectively. 30 On the contrary, in the AVADO study, the incidence of SB among anticoagulated bevacizumab-treated patients was 5% compared with 0% in anticoagulated placebo-treated patients, and 1.2% in the bevacizumab group that did not receive anticoagulants. 31

- Systematic reviews.

Data from 3,201 patients were collected. 32 Patients were allowed to continue with the study medication after an acute VTE event if the following criteria were fulfilled: absence of active bleeding, maintenance of stable anticoagulation for at least 2 weeks, and, in two of the three studies, absence of major vessel invasion. The SB rates, obtained from 194 anticoagulated patients, were similar in those treated with either bevacizumab or placebo (4.1 vs. 4.2%, respectively).

- Meta-analysis.

Finally, a meta-analysis including 10 studies and 6,055 bevacizumab-treated patients found that 10.5% of those who suffered a VTE and started anticoagulant therapy did not discontinue the antiangiogenic treatment. 33 In this subgroup, the bleeding rate was 1.9% (SB in 0.2% of cases), versus 1.2% among patients who did not require anticoagulant treatment.

#### Suggestions

- In the absence of bleeding, a reduction of the LMWH dose to be administered to a patient developing an acute VTE event while on antiangiogenic treatment is not suggested. Special caution is required in patients with central nervous system involvement.
- Resumption of the antiangiogenic therapy, after starting anticoagulant therapy for an acute VTE event, should be delayed for a reasonable period of 2 weeks to check the absence of any bleeding complication before adding any further risk factor. In case of life-threatening VTE, resumption of the antiangiogenic therapy is not recommended.

### Question 4: In Patients with CAT Requiring Surgery or an Invasive Procedure, When Should the Placement of an Inferior Vena Cava Filter Be Considered?

#### Background

The evidence supporting the use of inferior vena cava filters (IVCFs) in cancer patients is scarce. While some studies suggested that IVCFs are safe and effective, others found an increased risk of recurrent deep venous thrombosis (DVT; indeed, the cancer-related hypercoagulability is not corrected by the IVCF), as well as no benefit regarding pulmonary embolism (PE) incidence or short-term mortality. 34 35 36 37 38 39 40 Nevertheless, most guidelines recommend the use of IVCF in cancer patients with proximal acute DVT or PE when anticoagulant therapy is contraindicated. 41 42 43 44 Such is the case of major surgery or invasive procedures. Lumbar puncture, spinal anesthesia, or epidural catheter placement are considered as special procedures. 42 The use of IVCF would be particularly useful within the first 2 to 4 weeks after the acute thrombotic event due to the high recurrence risk in that particular period. 45 The use of IVCF is not clearly supported in other scenarios. 45 46 47 Furthermore, other potential adverse events associated with its use, such as placement or removal complications, migration, breakage, or thrombosis of the device, should be taken into account. 41 44 45 46 47

Moreover, anticoagulation should be restarted, and the IVCF removed, once the contingency that led to its placement is resolved. The strategy of IVCF removal should be defined prior to filter insertion. 41 43 44 45 46

#### Suggestions

- The use of a retrievable IVCF is suggested in cancer patients with acute proximal lower limb DVT or PE who require surgery or an invasive procedure that contraindicates anticoagulant therapy, particularly within the first 2 to 4 weeks after diagnosis. After 4 weeks from the diagnosis of the thrombotic episode, the placement of an IVCF is suggested in case of persistent proximal DVT (femoral or iliac veins).
- While the IVCF remains placed, the use of (at least) prophylactic LMWH, if not contraindicated by the bleeding risk, is suggested.
- Full anticoagulant therapy should be restarted and IVCF removed as soon as the cause leading to the placement of the filter is resolved.

### Question 5: In Patients with CAT Who Require Extended Anticoagulant Therapy beyond Six Months, What Is the Optimal Dose if LMWH Is Maintained?

#### Background

After completing 6 months of anticoagulant therapy for CAT, current clinical guidelines recommend to continue anticoagulation in case of active cancer and/or ongoing chemotherapy due to the high risk of recurrent VTE. However, since the observation period in available randomized trials comparing VKA and LMWH in this setting lasted 6 months, the drug/dose of choice for extended therapy is a matter of debate. 44 48

Useful data are provided by a subgroup analysis of an observational study, 49 and two single-arm prospective studies designed to evaluate LMWH safety over a 12-month period: the DALTECAN study, 50 in which dalteparin dose was reduced after the first month of treatment, and the TICAT study, 51 in which tinzaparin was used at full dose throughout the study.

Until stronger evidence becomes available, the dose of LMWH for extended therapy beyond 6 months should be tailored considering several issues: severity of VTE, cancer type and extension, ongoing anticancer therapies, bleeding risk, and patients' characteristics and preferences, which may change over time. 52 53 Indeed, intrinsic differences in the dosing of the various LMWH must be taken into account (as mentioned earlier, the standard therapeutic dose of dalteparin after the first month of treatment is 150 IU/Kg instead of 200 IU/Kg, while for the other molecules the full dose is maintained during the 6-month anticoagulation period).

Other options for extended therapy are VKA (although maintaining therapeutic INR ranges may be difficult in cancer patients under active antineoplastic therapy) and direct oral anticoagulants (DOACs). Very recently, two randomized clinical trials in patients with CAT have compared the efficacy and safety of edoxaban and rivaroxaban, direct factor Xa inhibitors, versus dalteparin using the CLOT trial scheme (200 UI/kg/day the first month, 150 UI/kg/day afterward) 54 55 56 for a minimum observation period of 6 months. In the Hokusai-VTE cancer study, no significant differences were observed in the rate of the primary composite endpoint (recurrent VTE and/or major bleeding), albeit patients in the edoxaban arm had a lower rate of VTE recurrence and a higher rate of major bleeding, especially gastrointestinal bleeding. 54 Similarly, the 6-month cumulative VTE recurrence rate was higher with dalteparin compared with rivaroxaban, although major bleeding was increased in the rivaroxaban arm. 56 The results of other ongoing studies may confirm a role of DOACs as an alternative for CAT extended therapy. However, current restraints by health authorities limit its use in patients with VTE in Spain and other countries, making LMWH the drug of choice for extended treatment of CAT.

#### Suggestions

- When LMWH treatment for CAT is to be prolonged beyond 6 months, the characteristics of each individual patient, of the underlying malignancy and its treatment and of the index VTE, should be considered to decide the optimal dose. Table 2 summarizes useful criteria to guide decisions.

**Table 2:** Criteria to decide LMWH dose when anticoagulant treatment for CAT is prolonged beyond the first 6 mo

	Full-dose LMWH a	Intermediate or prophylactic LMWH dose	LMWH dose increased 25%
VTE event	• Life-threatening symptomatic PE	• Incidental PE • Isolated lower limb DVT • Catheter-associated thrombosis	• VTE recurrence in spite of full-dose LMWH
Patient characteristics	• Obesity • Immobilization • Thrombophilia • Venous insufficiency, varicose veins, postthrombotic syndrome	• Renal impairment • Thrombocytopenia	
Neoplasm	• Metastatic disease • Tumor vessel compression • High thrombotic risk cancer: lung, pancreas, gastroesophageal	• Cancers with lower thrombotic risk: breast, prostate	
Cancer treatment	• Chemotherapy • Erythropoietin • Hormone therapy	• Immunotherapy • Targeted therapies	
Bleeding risk	Low: • No bleeding history	High: • Previous tumor bleeding • Previous bleeding history due to any other cause • Treatment with antiangiogenic drugs • Concomitant treatment with antiplatelet drugs	

Abbreviations: CAT, cancer-associated thrombosis; DVT, deep venous thrombosis; LMWH, low-molecular-weight heparin; PE, pulmonary embolism; SVT, superficial vein thrombosis; VTE, venous thromboembolism.

a For dalteparin, full doses after the first month of treatment, according to DALTECAN and CLOT studies, is 150 IU/kg/24 hours. 47 52

### Question 6: Should Anticoagulant Treatment Be Prolonged Beyond 3–6 Months in Cancer Patients with Catheter-Related DVT (CVC-DVT), When the Central Venous Line Is Maintained? Is LMWH Prophylaxis Indicated in Patients with previous CVC-DVT if a New CVC Is Needed?

#### Background

Some guidelines have addressed the initial management of central venous catheter (CVC)-DVT in cancer patients, although the strength of the evidence is limited. 43 44 57 58 59 In fact, the use of LMWH in this setting relies on general clinical trials of CAT. In general, anticoagulation for a minimum period of 3 months is recommended, even if the CVC is removed earlier. However, the CVC can be kept in place as long as it is functional, not infected, and DVT-related symptoms improve adequately.

In certain sense, if the CVC is maintained (or a new CVC is placed) beyond the first 3 to 6 months of anticoagulation for a CVC-DVT episode, the scenario could be comparable to a provoked VTE with a persistent risk factor. Therefore, the risk of recurrent thrombosis without anticoagulant treatment would be relatively high. In a study from the RIETE registry, the incidence of recurrent thrombosis after discontinuation of anticoagulant therapy in patients with CVC-DVT was 3.4 events/100 patient-years, but the proportion of patients in whom the central line remained inserted was not specified. 60 Another retrospective study suggested that after 3 months of LMWH, anticoagulation can safely be discontinued in patients with CVC-DVT when cancer is in remission and catheter is removed. 61 Two late recurrent VTE events (lower limb DVT) were observed in 16 patients in whom the central line was kept and continued anticoagulation beyond 3 months. In both cases, the patients were receiving only prophylactic doses of LMWH. No recurrent upper extremity DVT was observed in this group. Given the low number of patients and events, no definite conclusions regarding the optimal dose of LMWH for secondary prevention in this scenario can be derived.

#### Suggestions

- When the CVC is maintained after having completed 3 to 6 months of LMWH treatment due to a CVC-DVT event in a cancer patient, prolongation of LMWH therapy using intermediate or prophylactic doses is suggested. Treatment should be continued until CVC removal, as long as patient's bleeding risk is not high.
- In cancer patients with previous CVC-DVT history who require a new CVC, LMWH prophylaxis for at least 30 days after placement is suggested. Prophylaxis for a longer period, as long as the CVC remains inserted, may be considered, although patient's bleeding risk and preferences should also be valued.

### Question 7: How Should CAT Be Treated in Patients with Primary or Secondary Central Nervous System Involvement?

#### Background

Anticoagulation is effective, and usually well tolerated, in patients with gliomas 62 or cerebral metastases. 63 Nevertheless, some data may favor treatment modifications under certain circumstances:

- A retrospective study analyzed the outcomes of 364 patients with CAT, half of them with primary or metastatic brain tumors, for a median time of 6 months. 64 There were no differences between groups in the incidence of VTE recurrence (11.0 vs. 13.5 cases per 100 patients-year, *p* = 0.26) or major bleeding (8.9 vs. 6.0 cases per 100 patients-year, *p* = 0.80).
- Another retrospective study included 293 patients with cerebral metastases, 104 of who received therapeutic doses of enoxaparin due to acute VTE. 63 There were no differences in the 1-year incidence of cerebral hemorrhage compared with non-anticoagulated patients (total bleeding: 44 vs. 37%, respectively, *p* = 0.13). The risk of intracranial hemorrhage was fourfold higher for melanoma or kidney cancer when compared with lung cancer, although the increased risk was not associated with enoxaparin use.
- A meta-analysis with 1,480 patients with central nervous system (CNS) malignancies compared the risk of intracranial hemorrhage between those who received anticoagulant therapy with LMWH or warfarin, and those who were not treated with anticoagulant drugs. 65 The odds ratio (OR) of intracranial hemorrhage in anticoagulated patients was 2.13 (95% confidence interval [CI]: 1.0–4.56). The risk was not increased in patients with CNS metastases (OR: 1.07, 95% CI: 0.61–1.88), in contrast to those with cerebral glioma (OR: 3.75, 95% CI: 1.42–9.95). However, the higher incidence of intracranial bleeding did not seem to be associated with LMWH use (OR: 0.75, 95% CI: 0.24–2.33).
- Finally, since brainstem hemorrhages are particularly serious, any condition involving such area should be managed cautiously.

#### Suggestions

- In general, in the absence of other contraindications, in patients with primary or secondary neoplastic involvement of CNS, standard treatment of CAT with full-dose LMWH according to guidelines is suggested. However, the following exceptions may be considered:
  - In patients with secondary CNS involvement from melanoma or kidney cancer, and especially when the VTE event is not severe, a 25 to 50% reduction of the LMWH dose could be considered.
  - In cases of glioma in the brainstem, a 25 to 50% reduction in LMWH dose is suggested. If disease control with local treatment is achieved, a subsequent LMWH dose increase may be weighed.

### Question 8: Should Incidental Splanchnic Venous Thrombosis Be Treated?

#### Background

The most relevant information comes from a recent international registry promoted by the ISTH, although the study was not limited to cancer patients. 66 67 A total of 604 splanchnic venous thromboses (VTs), 177 (30%) of them incidental, were consecutively included. Sixty-two of 177 (35%) incidental splanchnic VTs were associated with nonhematologic cancer. In this latter group, one major bleeding event (1.2 cases per 100 patients-year) and seven thrombotic recurrences (8.1 cases per 100 patients-year) were observed during follow-up. 67 However, additional analyses may be useful for the decision-making process:

- The probability of being administered anticoagulant treatment was lower in both, patients with cancer and patients with incidental thrombosis. 67
- Treatments given to patients with incidental splanchnic VT were markedly heterogeneous regarding drug and duration. Patterns ranged from 6 months with parenteral anticoagulants, especially LMWH, to 24 months with oral VKA. 67
- Patients with thrombocytopenia (platelet count ≤100 × 10 ^9^ /L) were less prone to receive anticoagulant treatment, and showed the highest rate of major bleeding. 67 A thrombotic recurrent event was more frequently seen in male patients with incidental thrombosis and shorter duration of the anticoagulant therapy. 66
- Regardless of cancer, the rate of recurrences during anticoagulant treatment was similar in patients with symptomatic or incidental splanchnic VT. 67
- While on anticoagulant treatment, in patients with incidentally diagnosed splanchnic VT, the rate of major bleeding did not exceed that of recurrent thrombosis, although specific results in cancer patients are unknown. 67

In another recent study from the RIETE group including 521 patients with splanchnic VT, 309 (59%) incidental, most of them received anticoagulant therapy. 68 Compared with patients with symptomatic splanchnic VT, those with incidental splanchnic VT had a nonsignificantly higher risk of symptomatic VTE recurrence (hazard ratio [HR]: 2.04; 95% CI: 0.71–5.88) and a similar risk of major bleeding (HR: 1.12; 95% CI: 0.47–2.63). Active cancer was associated with an increased risk of recurrence (HR: 3.06; 95% CI: 1.14–8.17).

Although the quality of the evidence is low, international guidelines suggest that in cancer patients with incidental splanchnic VT, anticoagulant treatment should be considered in a case-by-case basis, taking into account clinical data suggestive of chronic thrombus, such as collateral circulation or portal cavernomatosis. 42 58 No recommendation about the need of an upper gastrointestinal endoscopy to look for esophageal varices that could be treated before starting anticoagulant therapy is made. There are no specific recommendations according to the splanchnic vein involved either. Nevertheless, anticoagulant therapy seems more warranted in patients with portal thrombosis candidates for liver transplantation, or in those with superior mesenteric vein thrombosis involving a large intestinal surface area.

#### Suggestions

- Unless contraindicated, in cancer patients diagnosed with incidental splanchnic VT, starting anticoagulant treatment is suggested.
- Treatment should be individualized in cases with clinical data suggesting chronic thrombosis, as well as in cases of isolated thrombosis of an intrahepatic portal segmental branch.
- Anticoagulant treatment should be maintained for at least 3 months.

### Question 9: In Cancer Patients with Acute VTE, What Platelet Count Threshold Would Imply Modifications in the LMWH Dose? Can Platelet Transfusions Avoid LMWH Dose *Reductions?*

#### Background

Full-dose anticoagulation with platelet counts higher than 50 × 10 ^9^ /L is universally accepted, also in the context of CAT. However, management with lower counts is controversial. Both, the ISTH in 2013 and the 2015 Canadian Consensus Guidelines recommended the following 41 69 :

- For VTE diagnosed more than 30 days ago, anticoagulant dose should be reduced in case of platelet counts lower than 50 × 10 ^9^ /L.
- In the acute phase of VTE (i.e., the first 30 days since onset), transfuse platelets to reach counts higher than 50 × 10 ^9^ /L, and anticoagulation should be kept at full therapeutic doses. This recommendation is based on the higher risk of recurrence during the first month after VTE diagnosis.

However, there are some concerns regarding this last recommendation 70 71 :

- First, sustaining an intensive platelet transfusion program to reach and maintain the threshold of 50 × 10 ^9^ /L is not easy and in many cases results unsuccessful.
- Second, transfusion may imply some safety concerns. In fact, an observational study showed that platelet transfusion aimed to reach counts greater than 50 × 10 ^9^ /L to maintain anticoagulation, was not only unable to reduce the hemorrhagic risk but was associated with frequent transfusion-related adverse effects. 72

By contrast, some studies assessed other alternatives:

- A recent observational study performed at the Memorial Sloan Kettering Cancer Center validated a dynamic strategy of enoxaparin dose reduction with the purpose of avoiding platelet transfusion. Such practice could be implemented at any VTE period, even in the first month. 73 In this study, therapeutic doses of enoxaparin were administered in case of platelet counts greater than 50 × 10 ^9^ /L, while half-dose was used with platelet counts between 25 and 50 × 10 ^9^ /L. Anticoagulant treatment was withheld if counts were less than 25 × 10 ^9^ /L. An IVCF was placed in 21 out of the 99 patients who participated in the study.
- Likewise, an intermediate strategy has also been proposed, which encourages platelet transfusion albeit with a lower threshold, 20 × 10 ^9^ /L. 71

In the very recent update of the ISTH guidelines, the experts suggest a dose modification strategy using 50% or prophylactic-dose LMWH for patients with platelet count of 25 to 50 × 10 ^9^ /L and acute CAT with lower risk of thrombus progression (i.e., distal DVT, incidental subsegmental PE, or CVC-DVT). In case of higher risk of thrombus progression, platelet transfusion to maintain a platelet count over 40 to 50 × 10 ^9^ /L and use of full-dose LMWH are recommended. 74

Finally, in a novel study from the RIETE registry (R. Lecumberri, MD, PhD, May 2018, unpublished data), the use of lower doses of LMWH in patients with acute CAT and severe thrombocytopenia seemed to be effective and safe, leading to low early rates of major bleeding and recurrent VTE, very close to those observed in cancer patients with normal platelet counts, although cancer-related mortality was significantly increased.

#### Suggestions

- In case of mild thrombocytopenia (platelet counts ≥50 × 10 ^9^ /L), keeping anticoagulant treatment at full therapeutic doses is suggested.
- In case of thrombocytopenia with counts lower than 50 × 10 ^9^ /L but higher than 20 × 10 ^9^ /L, a 50% reduction in the LMWH dose is suggested.
- In case of thrombocytopenia with counts equal or lower than 20 × 10 ^9^ /L:
  - If VTE was diagnosed more than 30 days ago, temporary interruption of anticoagulant treatment is suggested.
  - If VTE diagnosis was less than 30 days ago (acute VTE), platelet transfusion aimed to keep counts above 20 × 10 ^9^ /L, and anticoagulation using intermediate LMWH doses, is suggested.
- In the acute phase of VTE, placement of an IVCF can be considered in case of platelet counts equal or lower than 20 × 10 ^9^ /L or in patients with low cardiopulmonary reserve and counts ranging between 20 and 50 × 10 ^9^ /L, especially if thrombocytopenia is anticipated to continue for more than 5 to 7 days.

### Question 10: In Patients with CAT Requiring Anticoagulant Treatment and Who Were under Antiplatelet Therapy, When Should the Latter Be Maintained?

#### Background

Evidence on the need of maintaining or stopping antiplatelet therapy in patients with CAT is lacking. However, some data from atrial fibrillation (AF) and coronary artery disease patients may be useful:

- The use of VKA to treat a VTE event in patients with cancer is associated with a 3- to 6-fold higher hemorrhagic risk than that observed in patients without cancer. 75
- Many randomized clinical trials have shown that LMWH is safe and effective in acute coronary syndrome (ACS) without ST elevation. 76
- In AF patients who have stable coronary disease and for whom anticoagulation is indicated, oral anticoagulation therapy (mainly VKA) protects against ischemic stroke and coronary events. 77
- In AF patients who have stable coronary disease, the combination of VKA and acetylsalicylic acid (ASA), compared with VKA alone, does not reduce the risk of stroke or acute myocardial infarction but increases the risk of severe bleeding by 1.5- to 2-fold. 78
- Adding clopidogrel to the combination of warfarin and ASA in patients who have suffered an ACS markedly increases the rate of severe hemorrhage (4.6% at 30 days and 10.3% at 6–12 months). 79
- In patients treated for an ACS and in those who have undergone the placement of a coronary stent, the triple therapy consisting of oral anticoagulation, clopidogrel, and aspirin seems to be justified during a limited period. 80 In patients with high bleeding risk, the triple therapy might be limited to the first month after the ACS, and be followed by double therapy (VKA together with ASA or clopidogrel) for up to 1 year.

On the other hand, in the field of myeloproliferative neoplasms, both arterial and venous thrombotic complications are frequent. ASA is frequently used as primary prophylaxis or as secondary prophylaxis after an arterial event. In spite of the benefit of aspirin in reducing thrombotic complications, this benefit is probably overweighed by the increase of bleeding risk due to the association of ASA plus anticoagulation in comparison to anticoagulants alone. 81 82 83

#### Suggestions

- Since anticoagulation at therapeutic doses is effective to prevent coronary disease progression, and the addition of antiplatelet drugs increases the hemorrhagic risk, the indication of combined antiplatelet plus anticoagulant treatment for CAT should be limited to exceptional situations involving a very high risk of coronary event.
- Maintenance of antiplatelet therapy in patients who are going to start anticoagulant therapy for CAT is justified in case of recent (<1 year) ACS event or placement of a coronary stent.
- In patients with CAT carrying stents in other vascular beds, maintenance of antiplatelet treatment while on anticoagulant therapy should be decided in a case-by-case basis.

### Question 11: In Cancer Patients Treated with LMWH, When Should Anti-factor Xa Activity Be Monitored?

#### Background

In the pivotal studies comparing LMWH versus VKA in CAT, body weight–adjusted LMWH doses were used. 55 84 85 Data from those studies ruled out a significant LMWH accumulation over time, since anti-factor Xa activity (anti-Xa) remained stable. 86 Therefore, in spite of the higher risk of recurrence and bleeding in cancer patients, there is no evidence to support routine monitoring of anti-Xa activity to adjust LMWH dose.

- However, patients with severe renal failure (creatinine clearance <30 mL/min) were excluded from those clinical trials. Clinical practice guidelines suggest monitoring anti-Xa activity when using therapeutic doses of LMWH in patients with severe renal impairment, and also consider initial dose reduction when using enoxaparin or bemiparin. 87
- Due to variations in drug distribution, the suitability of monitoring anti-Xa activity in patients with extreme body weight and pregnant women has also been suggested. 87
- Additionally, monitoring anti-Xa activity in high bleeding risk scenarios, for instance, patients with thrombocytopenia, has also been proposed. 88 However, pharmacokinetics of LMWH would not be influenced under these conditions. The same applies to patients who have suffered a recurrent event in spite of treatment with LMWH. An empirical dose increase is recommended, although monitoring of anti-Xa activity might help in optimizing treatment.
- Importantly, the association between anti-Xa activity and either clinical efficacy or bleeding risk has not been undoubtedly demonstrated. 89 Therefore, in the earlier two mentioned scenarios, decisions on LMWH dose should not rely on anti-Xa assessment only.

Finally, the goals of anti-Xa activity for the different LMWH molecules have been retrospectively established. When LMWH is administered in a once-daily regimen, the goal of peak anti-Xa activity is generally around 1 IU/mL.

#### Suggestions

- In patients with CAT, routine monitoring of anti-Xa activity is not required to adjust LMWH dose.
- Renal function should be assessed in patients receiving LMWH at therapeutic doses. If creatinine clearance is less than 30 mL/min, LMWH dose adjustment according to peak anti-Xa activity is suggested (sample withdrawn 4 hours after subcutaneous LMWH administration). Repeated monitoring over time is advisable.
- Monitoring anti-Xa activity in patients with extreme body weight (after several days of treatment) and in pregnant women (once per trimester) is suggested.
- Monitoring anti-Xa in patients with thrombocytopenia or with other hemorrhagic risk factors is not suggested. The LMWH dose should not rely on this variable.
- Monitoring anti-Xa activity is not suggested for prophylactic doses of LMWH.

### Question 12: Should Thrombophilia Study Be Performed in Patients with CAT?

#### Background

Thrombophilia is mainly characterized by VTE at early ages (40–50 years), unprovoked events, or triggered by weak stimuli, recurrences, thrombosis at unusual sites, or strong family history of VTE. 90 The term “hereditary thrombophilia” usually includes deficiency of natural anticoagulants (antithrombin, protein C, protein S), factor V Leiden, and prothrombin G20210A mutation, while lupus anticoagulant or antiphospholipid antibodies are considered acquired thrombophilia. 90 The following points summarize some reasoning that does not support the search for these abnormalities in the context of CAT, since the clinical usefulness and benefits of such practice are rather limited or nonexistent.

- VTE management is generally guided by the clinical features of the event. Thrombophilic abnormalities do not usually change clinical decisions, except for antiphospholipid syndrome and antithrombin deficiency, associated with high recurrence risk, which may favor indefinite anticoagulant treatment. In fact, the main clinical practice guidelines on VTE management do not consider that thrombophilic abnormalities are relevant for initial treatment or duration of therapy. 43 91 92 Moreover, the selection of patients who would benefit from thrombophilia assessment is under discussion, although identification of thrombophilic abnormalities could influence decisions on anticoagulant treatment duration in patients with recurrent VTE or with strong family history. 93 94 95 96
- Patients with cancer exhibit a higher VTE risk. Although a thrombophilic factor could further increase the risk, VTE management in cancer patients is not influenced by the existence of associated thrombophilia: initial recommended treatment is similar to that used in nonthrombophilic cancer patients with VTE, and duration of therapy is mainly influenced by persistence of cancer and/or active oncologic treatment. 42 43 44 91

In sum, the existing literature does not provide evidence to justify, at first, a study of thrombophilia in patients with CAT, and guidelines do not recommend to perform it on a routine basis.

#### Suggestions

- The routine search for thrombophilia in patients with CAT is not recommended.

## Conclusion

VTE is an important and potentially avoidable cause of morbimortality in cancer patients that influences prognosis and quality of life. The variety and complexity of clinical scenarios in this setting explains why many therapeutic decisions remain controversial. This consensus was the result of the interest shown by three scientific societies—namely, SETH, SEOM, and SEMI—in CAT. The applied methodology allowed a multidisciplinary approach to each question, as well as validation of the final statements by a solid critical mass, which is particularly important in the absence of strong scientific evidence. The suggestions presented herein may constitute the bases for clinical decisions in specific complex circumstances, until these can be made leaning on reliable scientific evidence.
